# Comprehensive Rehabilitation for a Multilimb Amputee: A Case Report

**DOI:** 10.7759/cureus.83692

**Published:** 2025-05-07

**Authors:** Sandip Dhole, Maitreyi Patil, Rohit R Gaikar, Sumedh More

**Affiliations:** 1 Physical Medicine and Rehabilitation, All India Institute of Medical Sciences - Bibinagar, Bibinagar, IND; 2 Physical Medicine and Rehabilitation, Private Practice, Bengaluru, IND; 3 Physical Medicine and Rehabilitation, All India Institute of Physical Medicine and Rehabilitation, Mumbai, IND

**Keywords:** activities of daily living (adls), comprehensive rehabilitation, functional independence, multilimb amputation, prosthetic adaptation

## Abstract

Multilimb amputation poses a significant challenge to an individual’s mobility, independence, and overall quality of life. Comprehensive rehabilitation is essential for these patients to restore functional abilities and facilitate their reintegration into society. This case study details the rehabilitation of a 23-year-old male who sustained bilateral transradial and right transtibial amputations due to an electric burn injury. Upon presentation, the patient exhibited severe functional limitations, with complete dependence on caregivers for mobility, self-care, and activities of daily living (ADLs). A structured inpatient rehabilitation program was initiated, focusing on stump care, contracture prevention, range of motion, strengthening exercises, and functional training. He was educated on prosthetic options and fitted with customized prostheses, including a right cosmofunctional prosthesis with a hook terminal device, a left cosmofunctional prosthesis with a hand terminal device, a right patellar tendon-bearing above-knee prosthesis with supracondylar suspension, and an ankle-foot orthosis for his left foot’s equinus deformity. Rehabilitation emphasized upper limb prosthetic adaptation, progressive gait training, and stair-climbing exercises to enhance mobility and self-sufficiency.

At the time of discharge, the patient demonstrated significant improvement in functional independence. His Nottingham Extended Activities of Daily Living (NEADL) score increased from 0 to 27, out of a maximum score of 66, indicating partial autonomy. A follow-up assessment conducted three months later revealed continued progress, with his NEADL score reaching 47 out of 66, reflecting enhanced mobility and self-reliance. The NEADL scale, with scores ranging from 0 (complete dependence) to 66 (full independence), offers a comprehensive measure of functional ability across domains such as mobility, kitchen activities, domestic tasks, and leisure. The patient’s improvement from 0 to 47 illustrates a substantial functional gain and increasing independence in daily life.

This report highlights the importance of a structured rehabilitation program in promoting functional recovery and social reintegration in multilimb amputees. Early intervention, patient motivation, prosthetic rehabilitation, and a multidisciplinary approach play crucial roles in optimizing outcomes. The findings emphasize that with appropriate rehabilitation strategies, multilimb amputees can achieve a high level of independence, mobility, and meaningful engagement in their daily lives.

## Introduction

Multilimb amputation, involving the loss of both upper and lower limbs, is a rare but life-altering condition. Its exact prevalence is low, with studies estimating that multilimb amputations account for less than 1% of all limb amputations [1]. According to a study by Dillingham et al. (2002), the incidence of multiple limb loss in the US was approximately 0.3 per 100,000 persons annually [2]. The causes are often traumatic, such as high-voltage electrical burns, industrial accidents, or military injuries. Despite its rarity, the functional and psychosocial impact is profound, necessitating a coordinated, multidisciplinary rehabilitation approach.

Individuals with multilimb amputations encounter significant difficulties in their daily lives. One of the primary challenges involves the difficulty in donning prostheses, particularly for those who have lost both upper limbs. Limited mobility and high energy expenditure while using lower limb prostheses further hinder functional recovery. The inability to perform basic activities of daily living (ADLs), such as feeding, dressing, and personal hygiene, results in dependence on caregivers [3]. In addition to physical limitations, psychological distress, including depression and anxiety, is common among amputees. Research suggests that comprehensive rehabilitation programs that integrate medical, prosthetic, and psychological interventions significantly improve long-term outcomes [4].

Rehabilitation strategies for multilimb amputees focus on prosthetic adaptation, gait training, and social reintegration. Studies indicate that a structured rehabilitation program can lead to substantial improvements in mobility and independence. This case report explores the rehabilitation process and functional outcomes in a young adult multilimb amputee. It demonstrates the potential for significant recovery with a multidisciplinary approach that includes prosthetic training, functional exercises, and psychological support [5].

## Case presentation

**Figure 1:**
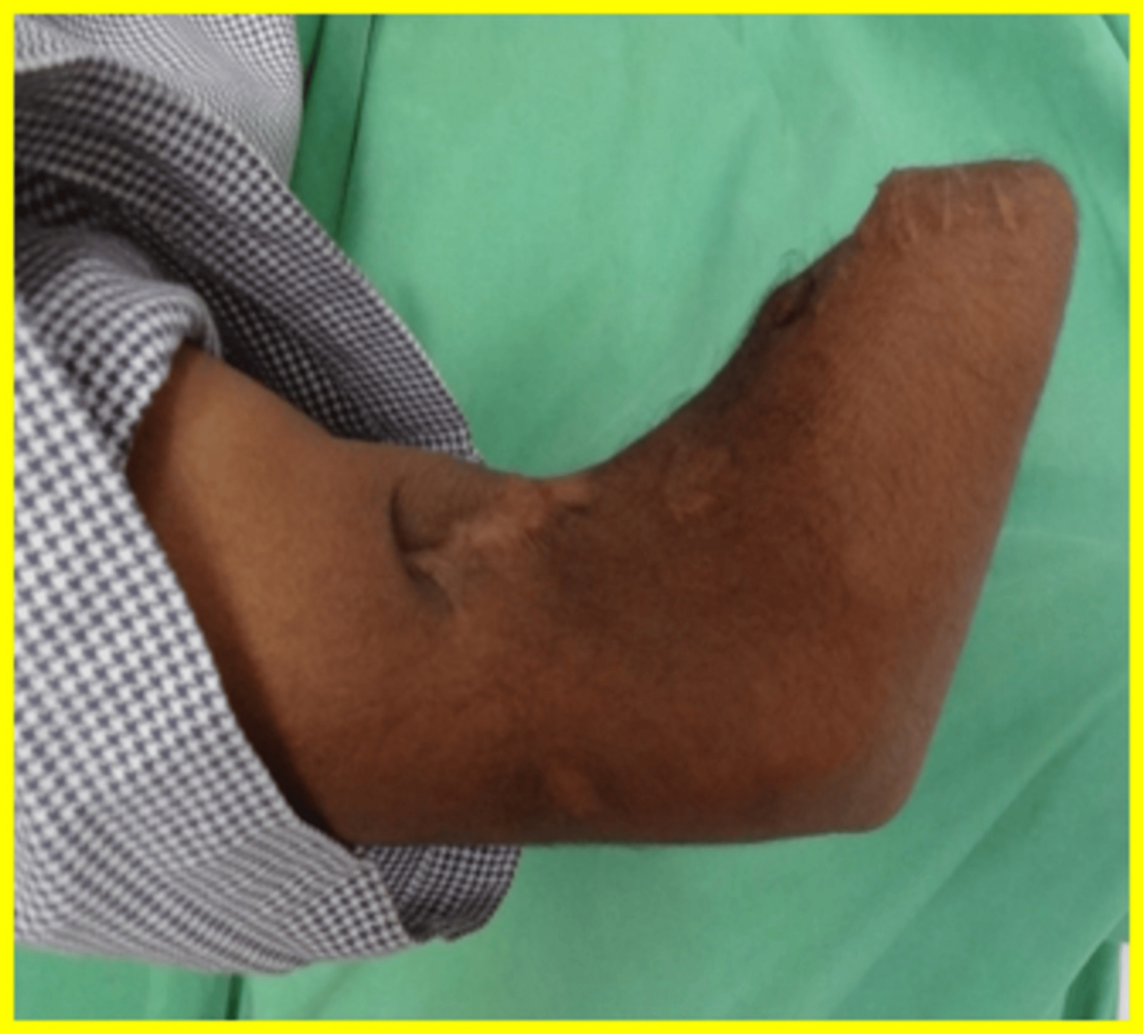
Right transradial amputation

A 23-year-old male, academically trained in Information Technology, presented nine months post-amputation following an electrical burn injury. The traumatic event had necessitated bilateral transradial and right transtibial amputations, while his left foot was affected by an equinus deformity. Upon presentation, the patient exhibited significant functional limitations, including the inability to perform basic mobility tasks, engage in self-care, or participate in domestic and leisure activities. Clinical examination of the right upper limb stump revealed a well-healed conical structure with a single bony prominence, measuring 10 cm in length and 20 cm at the proximal circumference, as shown in Figure 1.

**Figure 2:**
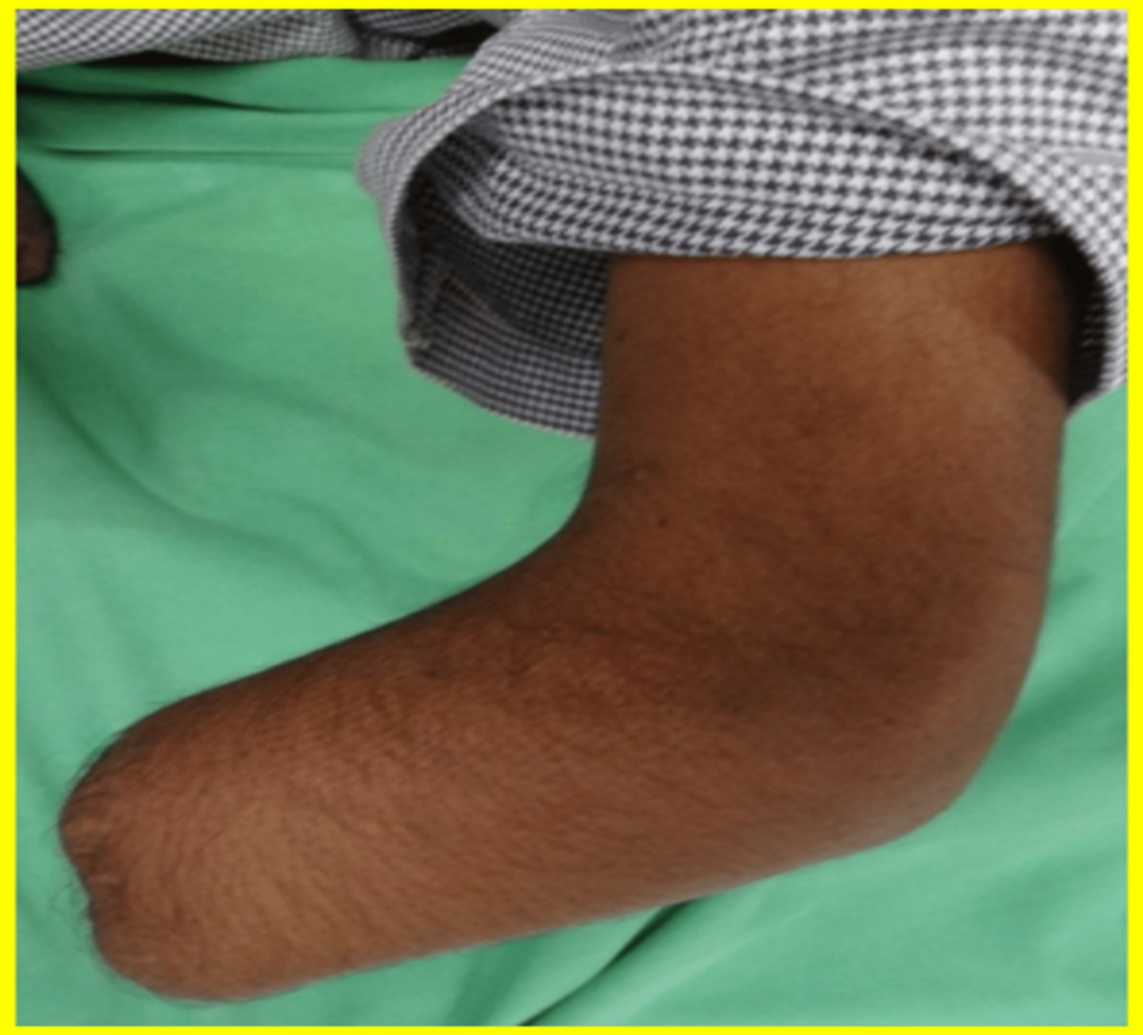
Left transradial amputation

The left upper limb stump displayed a similar conical shape, albeit with two bony prominences and a uniform circumference of 17 cm, as shown in Figure 2.

**Figure 3:**
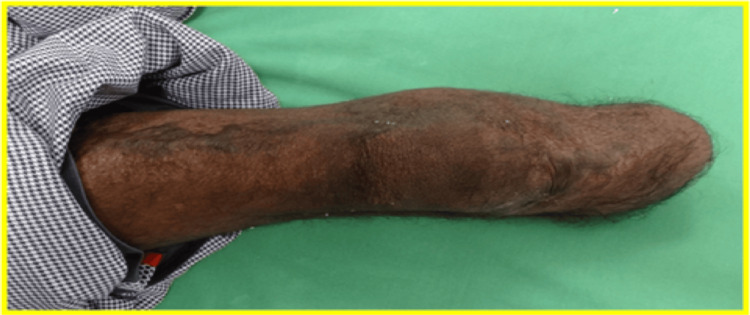
Right transtibial amputation

The right lower limb stump was cylindrical, well-healed, and free from bony prominences, with a length of 12.5 cm and a circumference of 25 cm, as shown in Figure 3.

**Figure 4:**
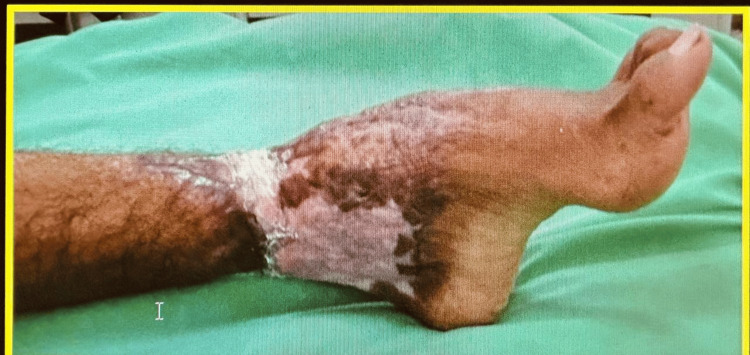
Left foot equinus deformity

His left foot exhibited a post-burn equinus deformity with a 45° fixed angle, which contributed to further mobility restrictions, as shown in Figure 4.

The Nottingham Extended Activities of Daily Living (NEADL) Index was used to evaluate the patient’s functional abilities. The initial assessment revealed a NEADL score of 0, indicating complete dependence on caregivers [6]. The patient was unable to walk, climb stairs, or move independently. Basic self-care tasks, such as feeding and dressing, were unmanageable, and he required assistance for all aspects of daily living.

Rehabilitation process

The rehabilitation program spanned 12 weeks and was divided into three structured phases with clearly defined dosimetry to address the patient’s complex functional deficits. The rehabilitation plan was designed to improve mobility, prosthetic adaptation, and overall independence. The program was structured into three phases:

Phase 1: Pre-prosthetic Training (Weeks One to Four)

Phase 1 involved daily therapy sessions, conducted six days a week, each lasting approximately 2.5-3 hours. The sessions included 30 minutes of stump care and skin inspection, 45 minutes of stretching and range of motion exercises (targeting shoulders, elbows, hips, and knees), and 1.5 hours of progressive resistance and isometric strengthening exercises for core stability, residual limbs, and proximal musculature, using TheraBands and body weight. Functional mobility training - such as bed mobility, assisted transfers, and seated balance tasks - was practiced for 30 minutes per session. The initial phase focused on preparing the patient for prosthetic fitting. Stump care and conditioning were emphasized to prevent skin breakdown and enhance tolerance for prosthetic use. Stretching and strengthening exercises were implemented to maintain joint flexibility and prevent contractures. The patient was also educated on available prosthetic options, enabling him to make informed decisions regarding his rehabilitation [7].

Phase 2: Prosthetic Prescription and Training (Weeks Five to Eight)

**Figure 5:**
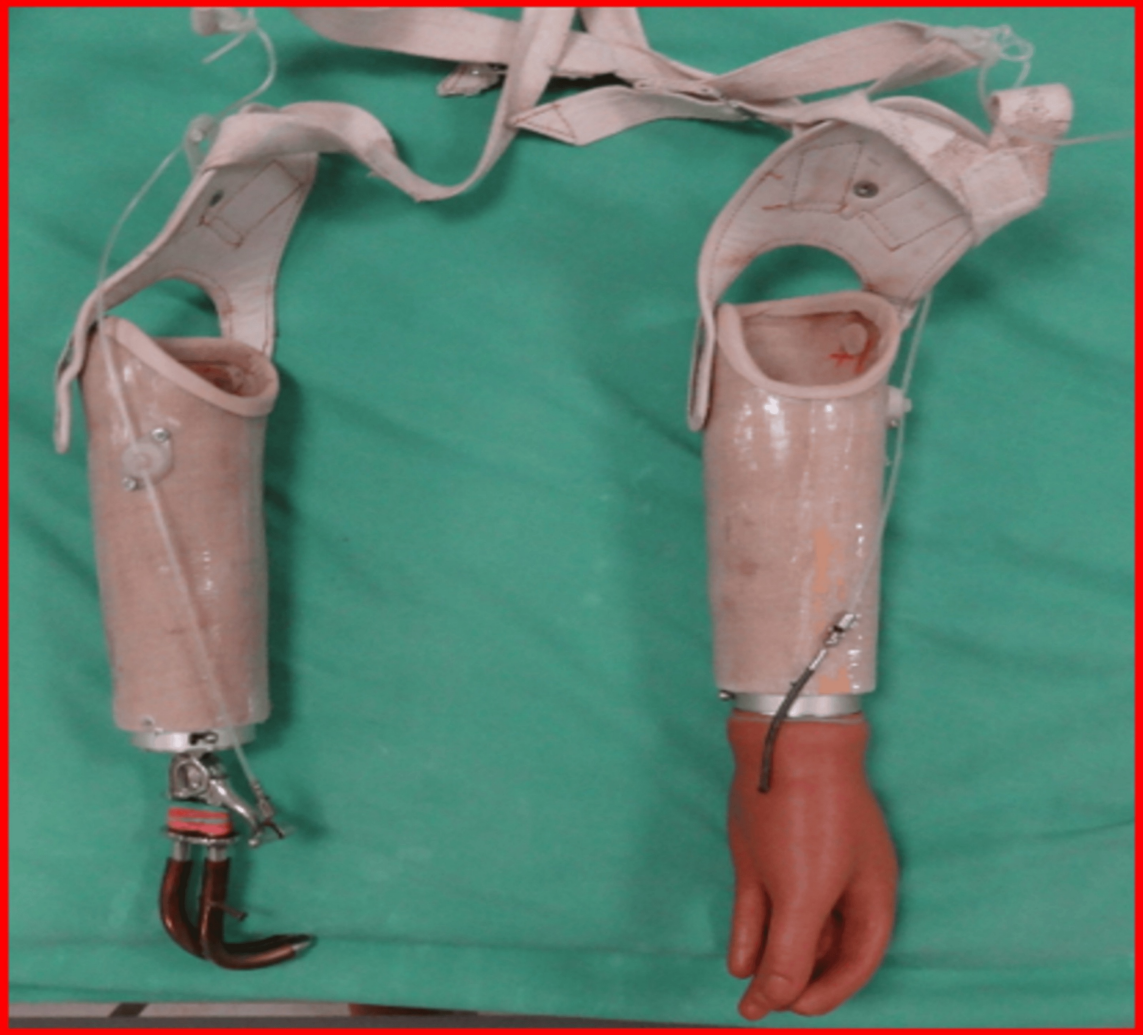
Right and left cosmofunctional prostheses - image 1 Right cosmofunctional prosthesis with hook as terminal device; left cosmofunctional prosthesis with hand as terminal device

**Figure 6:**
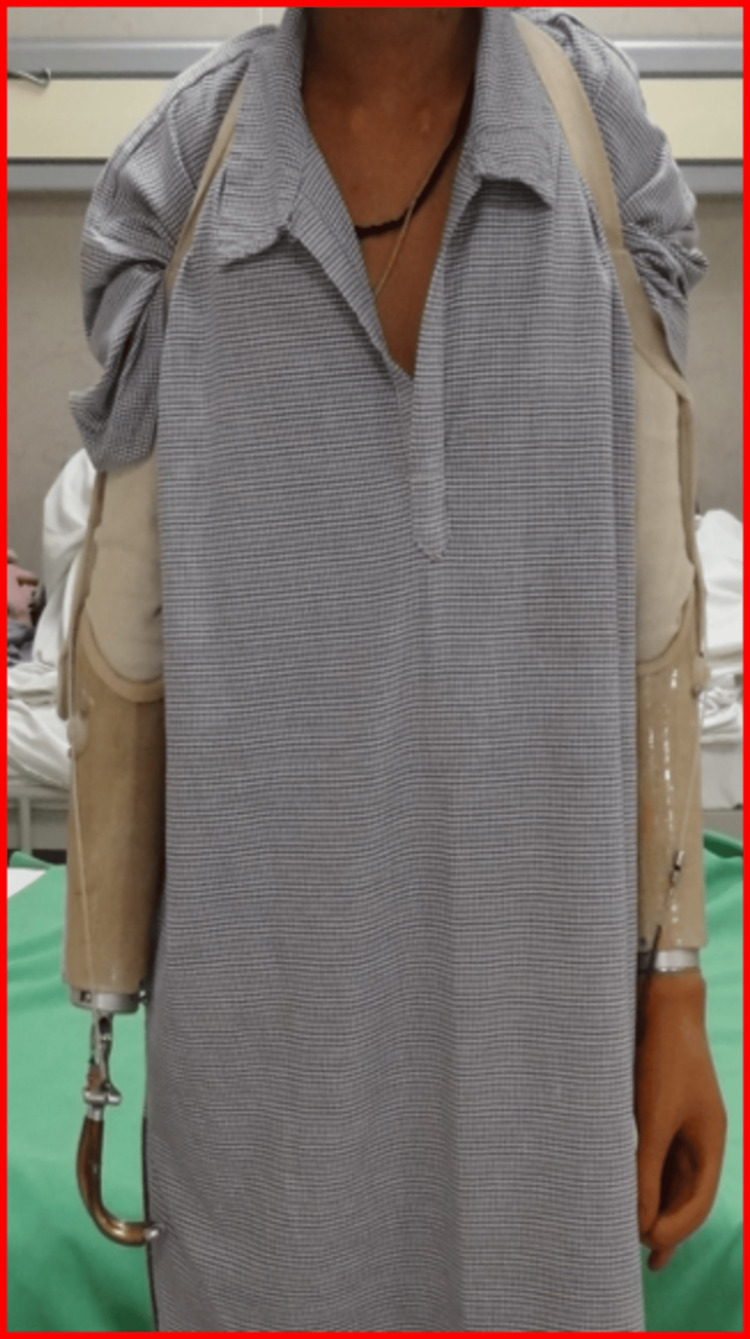
Right and left cosmofunctional prostheses - image 2 Right cosmofunctional prosthesis with hook as terminal device; left cosmofunctional prosthesis with hand as terminal device

After a comprehensive cardiopulmonary evaluation, a customized prosthetic prescription was developed. The patient was fitted with a right cosmofunctional prosthesis with a hook terminal device and a left cosmofunctional prosthesis with a hand terminal device, as shown in Figures 5, 6.

The training consisted of structured therapy five days per week, with three to four-hour sessions. Each day included 60 minutes of upper limb prosthetic functional use training (grasp/release, reaching, bilateral task practice), 60 minutes of gait training using parallel bars progressing to a walker, and 30 minutes of stair and obstacle navigation. Additional 30-minute sessions were dedicated to donning/doffing prostheses and residual limb hygiene. Gait training involved treadmill walking with body-weight support and static/dynamic balance exercises.

**Figure 7:**
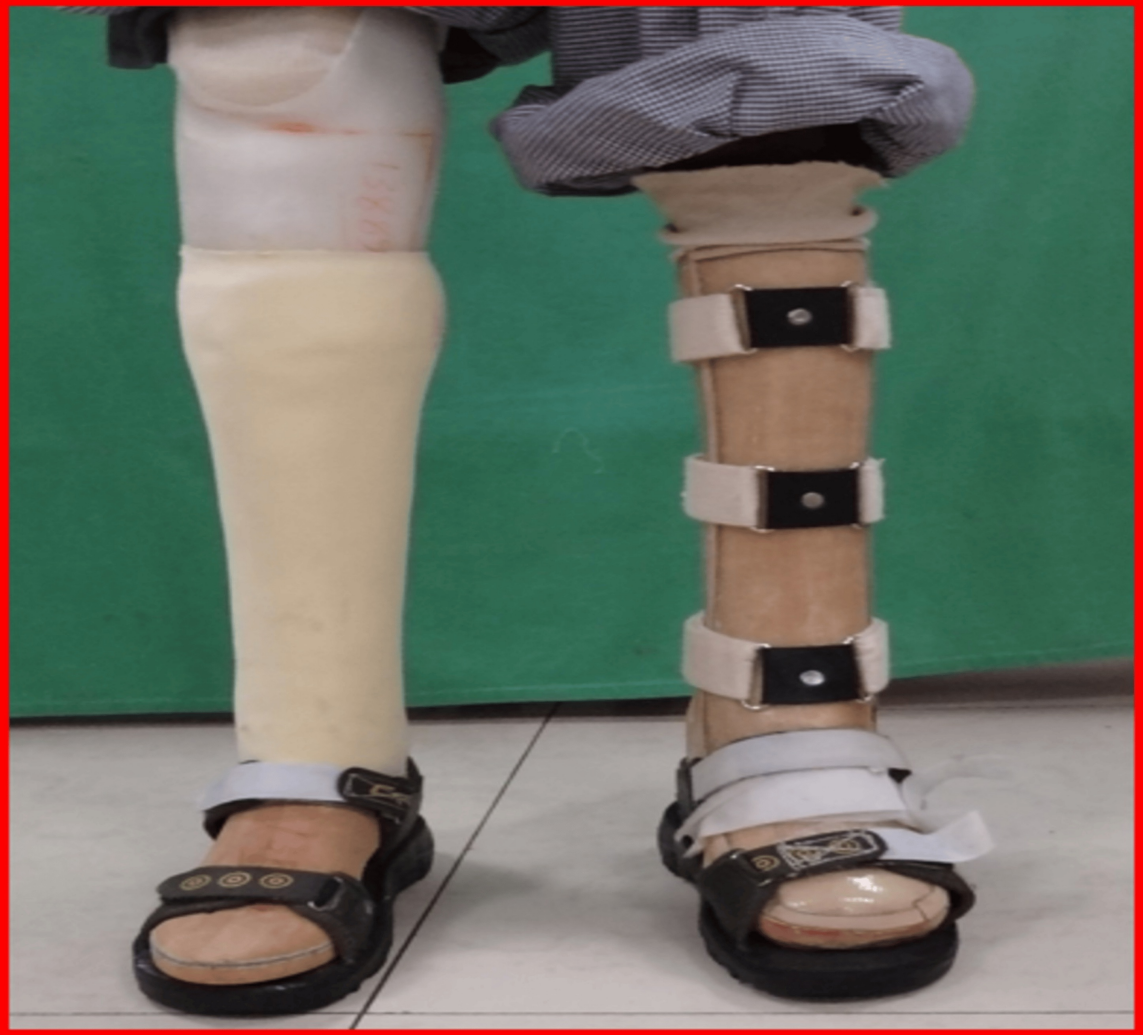
Right patellar tendon-bearing transfemoral prosthesis with supracondylar suspension; left AP shell AFO accommodating equinus deformity with marketed footwear AFO: ankle-foot orthosis; AP: anteroposterior

**Figure 8:**
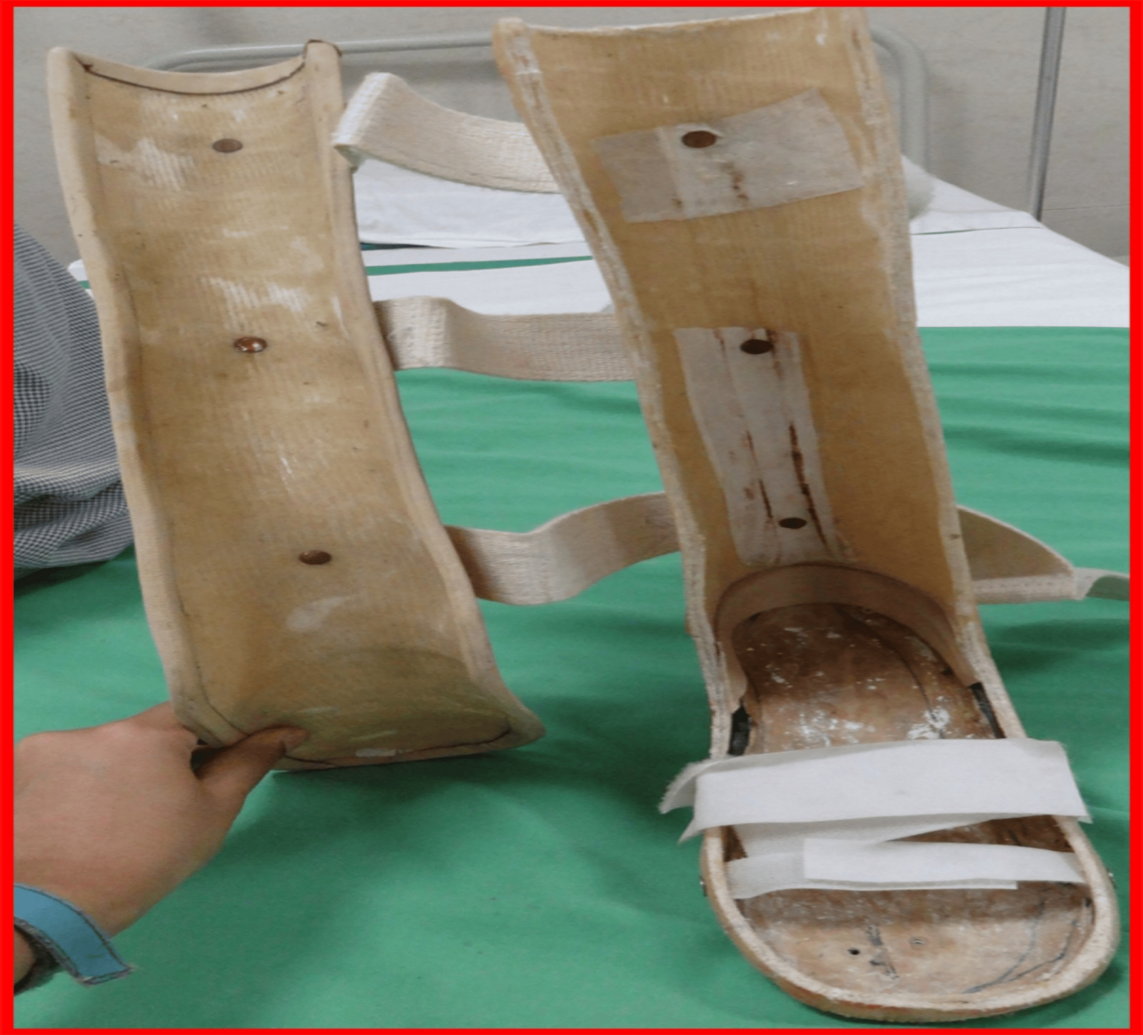
Left AP shell AFO accomodating equinus deformity AFO: ankle-foot orthosis; AP: anteroposterior

For lower limb support, the patient received a right patellar tendon-bearing prosthesis with supracondylar suspension. To accommodate his left foot’s equinus deformity, he was provided with an anteroposterior shell ankle-foot orthosis (AFO) and customized footwear, as shown in Figures 7, 8 [8].

Functional training commenced with upper limb prosthesis adaptation. The patient was trained in essential activities such as grasping, object manipulation, and using prosthetic hooks for daily tasks. Gait training was introduced in a stepwise manner, starting with support in parallel bars before progressing to independent ambulation. Stair-climbing training was also incorporated to enable unrestricted movement within his environment [9].

Phase 3: Social and Vocational Reintegration (Weeks 9-12)

In the final phase of rehabilitation, the patient underwent occupational therapy to enhance his ability to perform work-related tasks. Training in computer-based activities was provided to facilitate vocational rehabilitation. Community mobility training was introduced to help him navigate public spaces safely. Psychological counseling sessions were conducted to address emotional and social challenges associated with his condition [10]. This phase involved five-days-a-week sessions lasting two to three hours daily. The activities included 60 minutes of computer-based vocational rehabilitation tailored to the patient's background in IT, 30 minutes of community mobility and travel training (including simulated public transport use), and 45 minutes of advanced ADL and self-care tasks. Psychological counseling was provided twice weekly (45-minute sessions), focusing on adjustment, goal-setting, and coping skills. This phase also included environmental adaptation and family education sessions..

Outcomes and follow-up

At the time of discharge, the patient demonstrated significant improvement in functional independence. His NEADL score increased from 0 to 27 out of a maximum score of 66, indicating partial autonomy. A follow-up assessment conducted three months later revealed continued progress, with his NEADL score reaching 47 out of 66, reflecting enhanced mobility and self-reliance. The NEADL scale, with scores ranging from 0 (complete dependence) to 66 (full independence), offers a comprehensive measure of functional ability across domains such as mobility, kitchen activities, domestic tasks, and leisure. The patient’s improvement from 0 to 47 illustrates a substantial functional gain and increasing independence in daily life. This improvement reflected enhanced mobility and self-reliance [11].

The patient successfully transitioned to independent living. He manages a wine shop in Nashik and has expressed plans to establish a cyber cafe. His ability to sustain a livelihood demonstrates the positive impact of structured rehabilitation on occupational reintegration.

## Discussion

Prosthetic training plays a crucial role in the rehabilitation of multilimb amputees. Studies have shown that early prosthetic fitting prevents muscle atrophy and improves functional adaptation. Research by Radcliffe and Foort confirmed that customized socket designs enhance prosthetic comfort and usability [12]. Pezzin et al. have reported that individuals using functional prostheses, such as those with active terminal devices, demonstrated significantly greater independence in ADLs compared to users of cosmetic prostheses, primarily because functional designs allow for improved grasp, object manipulation, and task-specific utility, enabling users to perform self-care and vocational tasks more effectively [10]. Lower limb prosthetic adaptation is essential for mobility restoration. Studies by Murray and Fox demonstrated that patellar tendon-bearing prostheses improve weight distribution and reduce stump pressure sores [13]. The stepwise approach to gait training, as described by Jaegers et al., has been shown to enhance balance and walking speed in lower limb amputees [14].

Psychosocial support is another critical component of rehabilitation. Engel et al. emphasized that psychological counseling helps reduce post-amputation depression and promotes better prosthetic acceptance. Singh et al. found that early psychological interventions significantly improve long-term adaptation and quality of life [15]. Psychosocial support also helps address the emotional, cognitive, and behavioral challenges associated with limb loss, helping patients cope with altered body image, build self-efficacy, and improve adherence to rehabilitation goals. Our patient received structured psychological counseling twice weekly throughout the rehabilitation program, focusing on grief processing, motivation enhancement, and social reintegration, which contributed significantly to his functional and emotional recovery.

Long-term outcomes and employment prospects for multilimb amputees vary. Burger and Marincek reported that only 40-50% of amputees return to work, often due to limited accessibility and job modifications. The successful employment of the patient in this case highlights the importance of vocational rehabilitation in achieving long-term independence for multilimb amputees [16].

## Conclusions

This report demonstrates that multilimb amputees can achieve substantial functional independence through structured rehabilitation, early prosthetic fitting, targeted training, and psychological support. The combination of customized prosthetic solutions, multidisciplinary rehabilitation strategies, and social reintegration programs significantly enhances mobility and overall well-being. The findings underscore the need for individualized rehabilitation programs and advancements in prosthetic technology to further optimize recovery outcomes. Future research should focus on improving prosthetic acceptance rates, expanding access to advanced rehabilitation facilities, and integrating neuroprosthetic innovations for better long-term outcomes.
